# Cold exposure and human metabolism: A heterogeneous response across tissues and organs

**DOI:** 10.1080/23328940.2025.2599582

**Published:** 2026-01-04

**Authors:** Emily J. Tetzlaff, Curtis Hancock, Leander Waddell, Sheila S. Gagnon, Kari A. Mäkelä, Toni Karhu, Juha E. Peltonen, Karl-Heinz Herzig, Dominique D. Gagnon

**Affiliations:** a School of Kinesiology and Health Sciences, Laurentian University, Sudbury, ON, Canada; b Centre for Research in Occupational Safety and Health, Laurentian University, Sudbury, ON, Canada; c Human and Environmental Physiology Research Unit, School of Human Kinetics, University of Ottawa, Ottawa, ON, Canada; d Environmental Health and Safety, Global Services, Wenco International Mining Systems, Richmond, Vancouver, Canada; e Institute of Biomedicine, Medical Research Center, Faculty of Medicine, University of Oulu, Oulu, Finland; f Faculty of Sport and Health Sciences, University of Jyväskylä, Jyväskylä, Finland; g Sports and Exercise Medicine, Faculty of Medicine, University of Helsinki, Helsinki, Finland; h Helsinki Sports and Exercise Medicine Clinic (HULA), Foundation for Sports and Exercise Medicine, Helsinki, Finland; i Department of Gastroenterology and Metabolism, Institute of Pediatrics, Poznan University of Medical Sciences, Poznan, Poland

**Keywords:** Metabolism, cold exposure, muscle mass, sympathetic activation, organs

## Abstract

Cold-induced metabolic responses across human organs and tissues vary markedly and do not regulate metabolism uniformly. The magnitude and nature of these responses differ depending on the type of cold exposure, ranging from mild surface cooling and beta-adrenergic stimulation to deep tissue cooling impacting intracellular biophysical and metabolic properties. Upregulating brown adipose tissue (BAT) activity has been proposed to improve whole-body metabolism. Despite its high metabolic activity, BAT mass is typically only 50–100 g and may contribute less than 1% of total heat production during thermogenesis. In contrast, skeletal muscles and white adipocytes may play greater roles in thermogenic and metabolic regulation. Cold exposure triggers a cascade of metabolic responses across tissues, extending beyond fuel partitioning and the regulation of uncoupling proteins. It also alters gene expression, protein synthesis, and metabolic pathways. In response to cold, the body increases sympathetic nervous system activity, leading to peripheral vasoconstriction and energy substrate mobilization. Brown adipocytes increase mitochondrial uncoupling to produce heat, while skeletal muscle contributes through shivering and non-shivering thermogenesis. The liver adjusts glucose production and lipid metabolism, the heart and circulatory system adapt to altered hemodynamic demands, and the kidneys modify fluid balance. Endocrine systems, including the thyroid, amplify thermogenic capacity, and the brain integrates thermal sensing with behavioral responses. Cold exposure also modulates immune function, cytokine profiles and inflammatory pathways across tissues, and shifts in gut microbiome composition influence nutrient absorption, bile acid metabolism and energy homeostasis. These coordinated tissue-specific adaptations enable the maintenance of core temperature during cold stress.

## Introduction

Increasing metabolic activity in humans via cold exposure and brown adipose tissue (BAT) stimulation has been at the forefront of research to support the development of biomedical interventions to address metabolic disorders and obesity [1–5]. While some promising work has emerged, questions remain about the size and global importance of BAT in humans on whole-body metabolism in comparison to other tissues with a larger mass and metabolic contribution [6,7]. For example, knowledge of the concordant influence of BAT on whole-body metabolism and potential impact on the metabolic health of other metabolically active tissues, including human skeletal muscles and white adipose tissues (WAT) remains limited [8,9].

Human cold exposure is often considered an all-encompassing process rather than a progressive adaptation of physiological systems and responses to a changing stimulus. Based on both human and animal studies, exposure to a cold environment first activates skin thermoreceptors, leading to both sympathetic nervous system responses (e.g. vasoconstriction and BAT thermogenesis) and shivering thermogenesis, which is centrally regulated but not dependent on β-adrenergic stimulation [10–14]. Endocrine responses in humans, specifically β-adrenergic stimulation stemming from cold exposure, have been extensively investigated and are a key pathway in the regulation of peripheral vasoconstriction, heat production, and thermoregulatory responses to environmental stress to maintain thermal homeostasis [4,6,13,15–17]. As part of the β-adrenergic response to cold, rapid vasoconstriction and its redistribution of blood volume from peripheral tissue (i.e. skin) to core organs aim to preserve brain and cardiac functions for human survival [18,19]. However, the progressive and more profound tissue cooling of functional compartments and organs further alters intracellular biophysical properties and metabolic processes. These alterations include increased cytoplasmic viscosity, reduced enzyme reaction rates, and changes in oxygen-to-hemoglobin affinity, which may locally limit metabolic reaction rates [20–22]. These effects occur in parallel with increased whole-body energy expenditure, as thermogenic responses (i.e. shivering and non-shivering thermogenesis) elevate oxygen consumption and energy demand. From acute and surface cooling to prolonged and deep tissue cooling, it is critical to comprehensively assess the impacts of cold exposure on metabolic functions, as different cooling modalities will regulate physiological responses differently.

Cold exposure induces a cascade of metabolic responses across tissues, extending beyond fuel partitioning and uncoupling proteins. Gene expression, protein synthesis, and metabolic pathway regulation are all influenced by cold exposure. Multiple reviews on BAT and its implications on health and disease have recently been published *(Readers are directed to existing studies for detailed discussion of BAT thermogenesis* [6,23–29]). Yet, the role of more metabolically- and disease-relevant tissues in human metabolism, and their pathway activation. under cold exposure has not been sufficiently addressed. Investigating the metabolic regulation of larger tissues during cold exposure, from acute and surface cooling (β-adrenergic stimulation) to deep and prolonged cooling (biophysical and metabolic processes), may improve our understanding and elucidate novel pathways regulating metabolic disorders. Consequently, the present review summarizes the effects of cold exposure on metabolism and metabolic pathways in different human tissues, including skeletal muscles, white adipocytes, liver, kidney, heart, pancreas, bone, gastrointestinal tract, brain and metabolically relevant endocrine systems.

## Metabolic activity of organs and tissues

### Skeletal muscles

**Figure 1.**
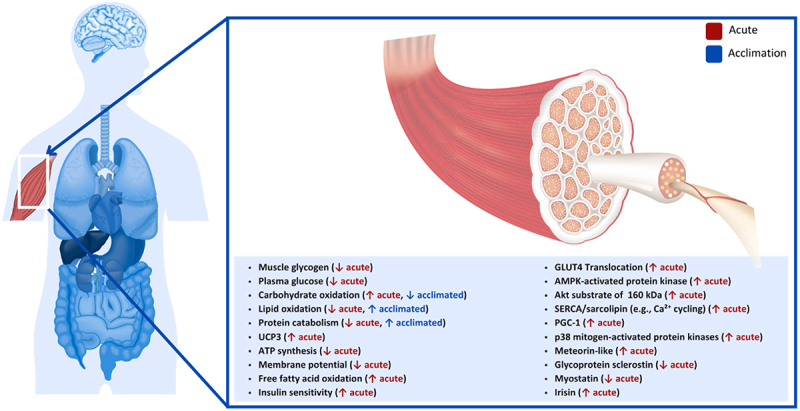
Effect of cold exposure on human skeletal muscle.

Skeletal muscles account for approximately 40% of total body mass in males and 30% in females and are the most significant tissue regulating metabolism and heat production in the human body (Figure 1) [30]. Muscular disuse, atrophy and other skeletal muscle abnormalities in humans have been associated with metabolic disorders, obesity, insulin resistance, and diabetes [31,32], and can significantly reconfigure local and whole-body metabolism. During cold exposure, skeletal muscles rapidly increase heat production, relying on various energy substrates through shivering and non-shivering thermogenesis mechanistic pathways. Shivering thermogenesis and sympathetic nervous system-mediated thermogenesis represent parallel and independently regulated outputs of the human body’s central-cold defense circuits [33–35]. These muscle contractions can occur from decreased skin and body core temperature, each neurally regulating 20–35% and 65–80% of the response, respectively (Note: in humans shivering can also occur in response to skin cooling without a measurable decrease in core temperature) [36,37]. Maximum shivering is reached when skin temperature is between 17–20°C and core temperature is 35°C [38]. During maximal shivering, an individual may reach five times their resting metabolic rate (i.e. 5–25 mlO_2_·kg·min^−1^), and in some cases, a value of 50% of maximal oxygen consumption may be reached [38]. These predicted values are supported by measured human data demonstrating comparable increases in whole-body oxygen consumption during sustained cold exposure [39].

Early work on human cold exposure and fuel source utilization investigated how skeletal muscle glycogen content is utilized to fuel shivering thermogenesis during 18°C cold-water immersion and with a body core temperature decreased to 35.5°C [40]. Lower muscle glycogen and plasma glucose concentrations following shivering suggested that intramuscular and systemic carbohydrates were essential sources of energy production. Extensive work has provided meaningful insights regarding fuel partitioning during shivering across intensities, muscle fibers, and cold acclimation [41–45]. Those studies have consistently demonstrated that as shivering intensifies, carbohydrates become the primary fuel source in humans, whereas energy production stemming from fat and protein sources is diminished [45]. Changes in fuel source utilization during prolonged cold exposure were also recently investigated in non-acclimated males [44]. During this study, participants were lightly clothed and placed in a chamber set at 7.5°C for 24 h, with thermal and metabolic responses recorded for 1 h at 6, 12 and 24 h [44]. Their findings showed that within the 6 and 12 h mark, carbohydrate oxidation was significantly reduced (119 to 51 mg·min^−1^; 30 to 10% $\dot{H}$_prod_) while lipid oxidation increased (105 to 141 mg·min^−1^; 55 to 80% $\dot{H}$_prod_), suggesting the finite sources of glycogen and glucose for energy production over prolonged periods to sustain shivering [44]. While glycogen and some circulating glucose are essential energy sources in humans during acute cold exposure, prolonged exposure increasingly relies on lipids and protein catabolism to preserve carbohydrate stores. When glycogen reserves are low, protein-derived substrates (glycerol and amino acids) contribute to maintaining blood glucose, highlighting the interplay among carbohydrate, lipid and protein metabolism during sustained cold stress [44].

Uncoupling proteins (UCPs) embedded within the mitochondrial inner membrane disrupt adenosine triphosphate (ATP) synthesis and membrane potential by leaking protons, mediating thermogenesis mechanisms. While UCP1 has been identified in BAT (discussed in later sections) [5,8], other isoforms potentially regulating metabolism have been identified in human and rodent skeletal muscles, including UCP3 [46–49]. UCP3 has been identified in many tissues, including BAT, skeletal muscles and the heart. Yet, its estimated muscle concentration remains low (1.7 pmol·mg^−1^), approximately 400 times lower than UCP1 found in BAT in mice [50], suggesting a limited direct role in thermogenesis. Nonetheless, UCP3 has been shown to lower ATP synthesis and membrane potential and increase fatty acid oxidation in humans [51], supporting its role as a marker of metabolic state in mice [46,48]. While some studies report that cold exposure does not influence UCP3 expression in skeletal muscles of mice [52], this does not exclude its involvement, as UCP3 activity may be regulated by post-translational modification, similar to UCP1 [50]. For example, evidence from knockout in mice indicates that UCP3 contributes to thermoregulatory and metabolic responses, though its precise role remains unclear [52]. Mice lacking UCP1 and UCP3 genes also had a more significant decrease in body temperature than UCP1 knockout alone. These findings support the need for further mechanistic studies of UCP3 in muscle non-shivering thermogenesis.

The sarcoplasmic/endoplasmic reticulum Ca^2 +^ -ATPase protein system (SERCA) in skeletal muscle is a key regulator of calcium homeostasis, primarily binding to Ca2+ ions in the cytosol and reuptake in the sarcoplasmic reticulum lumen, for muscle contraction and relaxation, and has been metabolically associated with most of non-shivering thermogenesis for heat production [53–56]. Its two main isoforms, fast-muscle isoform of the Ca^2+^-ATPase and the slow-muscle isoform, are transcriptionally regulated by triiodothyronine (T3) [57], a well-established hormone secreted during cold exposure and known to increase metabolic activity in humans and mice [58,59]. Among upstream signaling pathway regulating SERCA’s activity, cold-induced adrenergic stimulation and its activation of cyclic adenosine monophosphate and protein kinase A enhances calcium reuptake and ATP demand in mice [60]. The metabolic contribution of SERCA is still debated but some have estimated that it accounts for 42–48% of basal metabolic rate of mouse skeletal muscles and possibly close to 20% of total daily energy expenditure [61]. As an ATP-dependent process, its efficiency is heavily regulated by various proteins, including sarcolipin (SNL), known to uncouple Ca^2+^ transport from ATP hydrolysis, inhibiting SERCA activity, which in turn require greater ATP to pump Ca^2+^ back into the sarcoplasmic reticulum and generate more heat [62,63]. Despite its potential importance to whole-body metabolism and obesity, as well as its therapeutic potential for skeletal muscle pathologies, few animal studies were conducted to investigate SERCA and its protein regulators under cold stress. Studies using an SNL knockout model previously demonstrated the inability of mice to maintain body temperature during acute cooling [64,65]. Similar findings have been reported with a double knockout mice model for both SNL and UCP1 during acute cold exposure [66]. Interestingly, when using a UCP-1 knockout mice model only, the contribution of thermogenesis from non-shivering SERCA seems to be sufficient in maintaining heat production high enough to retain core temperature [67,68] and even increases its contribution to total heat production with repeated exposures in some mammals [69]. In humans, fewer reports are available; however, one study of patients with type II diabetes mellitus reported no changes in SNL expression following 10 days of cold acclimation, 6 h per day, in a chamber set at 14–15°C [70]. Interestingly, the same study observed that SNL expression levels did correlate with non-shivering thermogenesis (*r* = 0.893), but its relative contribution in relation to BAT non-shivering thermogenesis was unclear. Although the absolute contribution of SERCA to metabolic heat production in humans remains unclear, mechanistic studies of mice suggest it may play a role in thermogenic processes. Direct measurements in cold-exposed human studies are currently lacking, and further research is needed to determine its significance in human metabolism and thermoregulation.

Cold exposure lowers insulin secretion from pancreatic islets in rats [71] but increases insulin sensitivity in peripheral tissues, including skeletal muscles, for augmented glucose uptake, significantly increasing total body carbohydrates utilization [72,73]. Insulin functions as a vasodilator and counterintuitively works along cold-induced vasoconstriction upon cold stress, increasing glucose availability and its access to cells in both mice and humans [73–75]. The effect of a cold environment on insulin sensitivity primarily comes from increased glucose-transporter 4 (GLUT4) translocation to the plasma membrane of skeletal muscle fibers in both mice and humans [76,77]. GLUT4 is one of the primary glucose transporters in the human body and is believed to mediate much of the improved insulin sensitivity observed after acute bouts of exercise [78]. Cold exposure increases skeletal muscle GLUT4 translocation in humans via the sympathetic nervous system (SNS) activation and shivering-induced muscle contractions, which mimic exercise [79]. Interestingly, a recent report showed a postprandial glucose response 49% greater the day after an exercise bout in the cold compared to a thermoneutral environment [80], suggesting that acute cold exposure may enhance insulin sensitivity in recreationally active individuals, whereas subsequent days may impair glycemic responses differently. Future studies are needed to address the chronic effects of short-term cold exposure on insulin sensitivity.

The mechanistic theory behind the increase in GLUT4 is through the activation of AMP-activated protein kinase (an essential enzyme in activating both glucose and fatty acid oxidation), leading to an upregulation in phosphorylation of protein B kinase substrate 160 kDa (formerly known as TBC1D4), a known regulator of GLUT4 in mice and humans [81–83]. In animal studies, AMP-activated protein kinase was necessary to increase insulin sensitivity to stimulate glucose uptake in rat extensor digitorum muscles [81]. Insulin-stimulated muscle glucose uptake was also shown to be impaired in rats with a diet-induced deficit of protein B kinase substrate of 160 kDa (through a high-fat diet) compared to those with normal levels. This effect was slightly rescued by exercise; however, the rats without protein B kinase substrate of 160 impairment still had higher insulin-stimulated muscle glucose uptake [84]. Cold exposure seems to differ in its effects on GLUT4 from exercise slightly. However, increased skeletal muscle GLUT4 was observed in type II diabetics after 10 days of cold exposure, which could not be explained by an increase in AMP-activated protein kinase [70]. One proposed contributor to increased GLUT4 expression during cold exposure is SNS stimulation of β-adrenergic receptors on various tissues; however, this mechanism has not yet been fully explained [70]. Although SNS activation may influence glucose uptake in some tissues in humans an mice, particularly via adrenergic signaling, it is unlikely to be the sole driver in skeletal muscle, where other pathways (e.g. insulin sensitivity) likely play more dominant roles [79,85].

β-adrenergic stimulation under cold exposure activates mechanisms involved in adaptive thermogenesis in skeletal muscles, including the expression of peroxisome proliferator-activated receptor-γ coactivator-1α (PGC-1α), a co-transcriptional regulating factor responsible for the transcription of nuclear respiratory factors and mitochondrial transcription factor A, known to regulate cellular growth and mitochondrial functions in humans [86–88]. Examining PGC-1α messenger ribonucleic acid expression and regulating pathways in animal studies revealed increased AMP-activated protein kinase, p38 mitogen-activated protein kinases, and PGC-1α during cold exposure ranging from one to 12 h [89–91]. It was reported that there is an increase in the expression of PGC-1α of the magnitude of 15-fold following 3 h of cooling at 4°C [89]. In humans, the effects of 10 min of lower limb immersion in 8°C cold water on PGC-1α messenger ribonucleic acid of the vastus lateralis were investigated, and a 1.3-fold increase 3 h post-cooling was found [92]. While the magnitude of change between animal and human studies seems to differ, many prospective factors have yet to be explored to explain those differences. Notably, the intensity of sympathetic activation and varying concentrations of circulating catecholamines under varying homeostatic challenges have yet to be addressed. The selection of tissue to be analyzed may also introduce variability as mitochondrial biogenesis pathway stimulation is known to differ between muscles in mice [90]. Nonetheless, cold exposure, similarly to exercise, appears to induce a metabolic cascade initiated with an increase in the expression of p38 mitogen-activated protein kinases and AMP-activated protein kinase, including upstream regulators of PGC-1α, the upregulation of PGC-1α, as well as potential downstream mitochondrial biogenesis factors such as nuclear respiratory factors, and mitochondrial transcription factor A.

Moreover, skeletal muscle tissue and its endocrine functions secrete numerous myokines, including many involved in whole-body metabolism in humans and mice [93–95]. Meteorin-like is a recently discovered myokine that is stimulated by both exercise and cold exposure and is associated with WAT beiging and browning, energy expenditure, and systemic inflammation [93–95]. Increased levels of meteorin-like in mice have recently been linked to glucose metabolism in skeletal muscles via the calcium-dependent AMP-activated protein kinase catalytic subunit alpha 2 pathway, resulting in the increased transcription of GLUT4 and, ultimately, higher glucose uptake [96]. While studies focused on the effects of meteorin-like on human metabolism remain sparse, a recent study claimed that the levels did not significantly change after ice swimming [97]. Still, the glycoprotein sclerostin decreased significantly after cold exposure [97,98]. Sclerostin, an inhibitor in canonical wingless/Integrated pathways involved in developing and maintaining tissues [99,100], plays a vital role in human and mouse bone homeostasis [100–102]. A lack of sclerostin expression has been associated with an increase in bone production [100,101] and is the cause of the high bone mass human syndromes Van Buchem disease and sclerosteosis [100,101]. As the dysregulation of sclerostin expression can lead to bone loss [100,101], sclerostin has been identified as a molecular target to treat many bone-related diseases in humans, including osteoporosis [99–101,103], Paget’s disease of bone (PDB) [99], multiple myeloma bone disease [104], osteonecrosis [103], and bone tumors [103]. To differing degrees of involvement and certainty, sclerostin has also been suggested to be involved in chronic kidney disease [99,105], diabetes [99,106], rheumatoid arthritis [103,105], anorexia nervosa [107], metabolic disease [106] and oral inflammatory diseases [108].

Furthermore, myostatin, a growth factor responsible for skeletal muscle development and remodeling, has decreased (−18.2%) serum concentration levels in healthy males following 10 whole-body cryotherapy sessions (3 min at −110°C) [109]. Decreasing blood concentrations of myostatin in a sample of students were also observed following 12 resistance training sessions, followed by 3 min of cryotherapy at −110°C, from 4.7 to 3.8 ng·mL^−1^ [110]. Although body cooling may seem favorable to induce lower expression of myostatin with associated muscle growth, the changes in biophysical properties of skeletal muscle cells upon cooling (i.e. lower metabolic activity), combined with exercise, may compromise muscle growth responses. Some studies have observed no differences in myostatin circulating levels or gene expression following exercise and cold stress in humans [111–113]. In contrast, other studies of active males have found lower physical performance on one rep max (RM) tests following resistance training and post-exercise cold-water immersion [114,115]. This suggests that lower expression of growth factors such as myostatin under cold exposure may be beneficial. Still, a more comprehensive spectrum of growth factors would likely reveal a detrimental impact of body cooling on muscle development and remodeling [112].

Interestingly, the downregulation of MTSN during cold stress has been associated with increased irisin levels in mice [116]. In humans, irisin is known to regulate genes related to muscle growth and adipocyte differentiation and further impacts metabolic pathways, including the tricarboxylic acid cycle and independent substrate metabolism [117]. Although direct energy expenditure outcomes of irisin fluctuations and its stimulation under cold exposure have yet to be quantified, early promises of its action on adipocyte browning, obesity, insulin resistance and type II diabetes were investigated. Much ambiguity concerning circulation and detection levels remains regarding this myokine, as many investigations of humans and animals relied inadequately on tested polyclonal antibody techniques rather than mass spectroscopy to determine its concentration in biological samples [118]. Nonetheless, based on animal interventional studies and human clinical studies, skeletal-muscle-secreted irisin seems to metabolically modulate not only skeletal muscles but numerous tissues under cold stress, including adipocytes and hepatocytes, as well as the brain, the pancreas and kidneys [119].

**Table:** 

**Key Highlights of the Effect of Cold Exposure on Human Skeletal Muscle** Shivering thermogenesis is the primary acute defense; energy expenditure can reach ~5× resting levels. Fuel use shifts over time: glycogen and glucose dominate acutely, while lipids (and protein catabolism) sustain prolonged cold. Molecular thermogenesis pathways include UCP3 (fat oxidation, limited direct heat role) and SERCA/sarcolipin (Ca^2 +^ cycling, potential non-shivering contribution). Insulin sensitivity is enhanced through GLUT4 translocation (SNS- and AMPK-mediated), improving glucose uptake in muscle. Cold-induced myokines (e.g. meteorin-like, irisin, sclerostin) link skeletal muscle to WAT browning, glucose metabolism, and bone regulation. Exercise-cold interactions may lead to myostatin suppression and affect muscle remodelling. Given its mass, skeletal muscle is the dominant contributor to systemic metabolic and thermogenic adaptation to cold.

### White adipocytes

**Figure 2.**
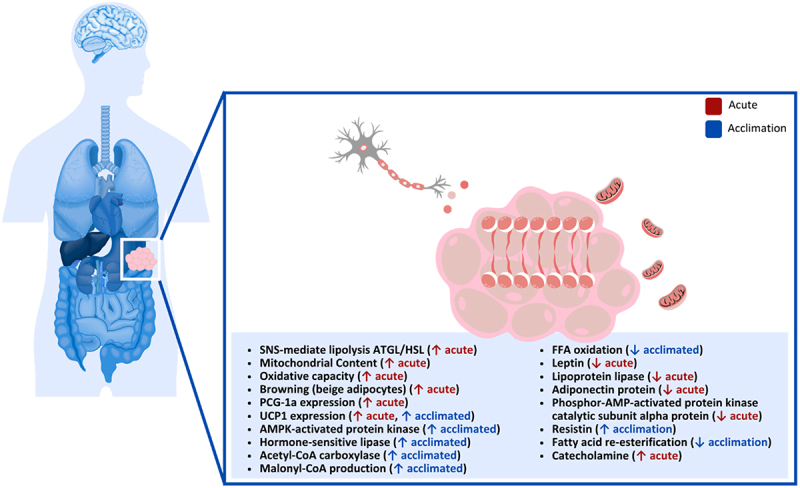
Effect of cold exposure on human white adipose tissue.

WAT is a highly dynamic endocrine organ that plays a central role in energy metabolism and control of glucose and lipid homeostasis (Figure 2). White adipocytes are the predominant type of adipose tissue in the human body and are the primary tissue for energy storage. In humans, the major WAT depots are located under the skin (i.e. subcutaneous, inguinal fat) and intra-abdominally, surrounding the internal organs (i.e. viscerally; gonadal, mesenteric, omental and perirenal fat). When energy is in surplus, in both humans and rodents, WAT stores energy primarily in triacylglycerol and releases free fatty acids and glycerol under conditions of negative energy balance [120]. During cold exposure, lipolysis mobilizes triacylglycerol-derived fatty acids from WAT to meet whole-body energy demands, and reliance on lipid substrates may increase may increase further when glycogen reserves are limited in humans and animals [120,121]. White adipocytes also secrete peptide hormones and adipokines, which can act in an autocrine, paracrine, and/or endocrine manner to regulate whole-body energy homeostasis [120,122].

WAT depots and their lipolytic activity are sympathetically innervated, as noradrenaline release is key for controlling lipolytic rates and regulation of hyperplasia (cell proliferation) in rodents [123–125]. However, WAT depots in mice display depot-specific characteristics, with visceral WAT more innervated and vascularized than subcutaneous WAT, and both substantially less than BAT [126]. Consequently, cold exposure may elicit milder gene expression changes in WAT compared to BAT in animals [127,128]. In humans, cold-mediated activation of the SNS stimulates β-adrenergic receptor signaling in both WAT and BAT to activate cellular lipolysis [79]. In WAT, β-adrenergic stimulation mobilizes free fatty acids to be utilized by thermogenic organs, such as skeletal muscle and BAT [4,79]. In humans, mild cold stimulus activated WAT intracellular lipolysis, with activity associated with BAT metabolic activity but not muscle-shivering [79]. Therefore, SNS-mediated WAT lipolysis and BAT thermogenesis are closely integrated processes [79].

While β3-adrenergic receptors are the predominant β-subtype in rodent adipose tissue, only 20% of β-adrenoceptors in human WAT are β3-receptors, inducing a limited lipolytic or thermogenic response in the cold [120,129]. Conversely, in both humans and animals, responses are primarily mediated by β1- and β2- adrenergic receptors [120,130–132]. As a result, while both adrenaline and noradrenaline are essential for increasing glycerol and free fatty acid secretion from WAT [129,133], circulating adrenaline may be of greater importance in humans due to the more significant role of β2-receptors, which are more sensitive to adrenaline [129].

WAT has been shown to acquire characteristics of BAT via browning, where adipocytes can increase mitochondrial content, oxidative capacity, and thermogenic ability in mice [134,135]. In humans and mice, brown adipocytes in WAT, or brite or beige adipocytes, arise as multilocular adipocytes during adrenergic stimulus [136,137], cold exposure [138], or exercise [139,140]. However, brite adipocytes are less understood and characterized than BAT and WAT. When mice are exposed to a cold stimulus, the development of brown adipocytes and browning of WAT depots occurs, increasing the thermogenic capacity in WAT depots [126,141,142]. In mice, browning of WAT occurs to a greater degree in subcutaneous WAT compared to the visceral depots [126,143]. However, humans display an opposite pattern in gene expression, with a higher browning gene expression in visceral WAT than in subcutaneous WAT [144]. Therefore, the interpretation of murine rodent studies on the browning of WAT should be considered very cautiously.

Regional and depot-specific differences in catecholamine-mediated lipolysis and triacylglycerol turnover rate in human WAT have been well-documented [145–147]. Regionally, abdominal depots show significantly greater lipolytic responses than gluteal depots. Internally located visceral depots (epididymal and retroperitoneal WAT) also exhibit more significant lipid mobilization than the more externally located depots (i.e. subcutaneous WAT) [120,121,148,149]. However, cold exposure increases energy demand and drives a higher noradrenaline turnover in inguinal and retroperitoneal WAT than in epididymal WAT [121,150].

Subcutaneous WAT in humans can upregulate UCP1 and other mitochondrial genes in response to acute cold exposure [142]. The primary stimulator of UCP1 synthesis and activation, as well as WAT lipolysis, is norepinephrine [121,151] Cold exposure has also been shown to increase the expression of PGC-1α in adipose tissue [152–154]. A study that observed seasonal changes in WAT mitochondrial gene expression found that UCP1 and PGC-1α expression were higher in adipose tissue biopsied during colder months than warmer months [142]. There is evidence that human adipocytes may have cold receptors, such as transient receptor potential melastatin 8 [155], more controversially TRPA1 [156], along with other cold-sensitive ion channels [157,158], such as K2P channels like Kcnk3 [159], epithelial sodium channel (ENaC) [160], and TRPC5 [161]. These receptors generally activate UCP1 expression, discovered when adipocytes were studied *in vivo* [155,162,163]. AMP-activated protein kinase and hormone-sensitive lipase were reportedly elevated in abdominal subcutaneous WAT biopsied in the colder months, suggesting that there are also seasonal changes in human thermogenic gene expression in WAT [142]. Although this has not been observed consistently, a limited role of seasonal cold exposure was reported in humans on WAT UCP1 regulation [164]. Nonetheless, acetyl-CoA carboxylase (AAC) was also reported to be elevated, which produces malonyl-CoA and inhibits the oxidation of fatty acids in the mitochondria. As such, thermogenic activity in WAT via brite adipocytes may have a greater reliance on glucose metabolism, as previous research has shown that brite adipocytes positively affect glucose metabolism [165]. However, the increased UCP1 expression and brite adipocyte thermogenic capacity acquired during cold exposure can be reversed when returned to warmer temperatures, as demonstrated in mice [166]; this may explain differences in seasonal WAT responses. Acute cold exposure (30 min) also generated changes in gene expression of UCP1 and PGC-1α in human WAT [142]. However, when acute cold exposure occurred during the colder months, no additional upregulation of thermogenic genes in WAT was observed [2,142].

WAT integrates metabolic signals and responds to environments by synthesizing endocrine factors called adipokines [167]. Sympathetic stimulation during cold exposure leads to a rapid decrease in the gene expression and levels of leptin and lipoprotein lipase in mice [168–170]. Other energy-metabolism-related adipokines released from WAT in mice include adiponectin and resistin [167]. In a study examining adipokine responses to cold acclimation in rodents, one day of cold exposure decreased adiponectin protein in serum and its messenger ribonucleic acid levels in WAT [171]. Acute cold exposure also reduced adiponectin levels in healthy males in as little as 30 min, compared to non-cold exposed males [172]. The decrease in adiponectin was coupled with decreased phosphor-AMP-activated protein kinase catalytic subunit alpha protein levels and other enzymes involved in oxidative metabolism, suggesting that, like in mice, human WAT relies on glycolytic strategies during the initial stage of cold exposure [167]. It should be noted that these responses were reversed after three days of cold exposure, restoring/increasing adiponectin and AMP-activated protein kinase catalytic subunit alpha signaling toward WAT lipid oxidation [167]. Alternatively, resistin protein concentrations in mice increased during the first seven days of cold acclimation, inhibiting fatty acid re-esterification and increasing their release from adipocytes to supply other tissues with fatty acids [167,173]. However, resistin concentrations observed in rodent WAT are significantly higher than expression in humans, and its role in metabolism is, therefore, likely very different [174,175].

In summary, cold exposure increases catecholamine turnover in WAT, leading primarily to an increase in lipolysis to fuel other thermogenic organs. Browning of WAT can also occur with cold exposure, increasing its thermogenic capacity by increasing mitochondrial content and proteins involved in the breakdown of metabolic substrates. However, thermogenic adaptations disappear when returned to a warmer environment, such as with seasonal temperature changes.

**Table:** 

**Key Highlights of the Effect of Cold Exposure on Human White Adipose Tissue** Cold stimulates SNS-mediated lipolysis, mobilizing fatty acids and glycerol to fuel BAT and muscle thermogenesis. Browning of WAT occurs with cold, though responses differ (i.e. stronger in rodents, depot- and season-dependent in humans). Cold-sensitive ion channels may directly regulate adipocyte thermogenic gene expression. Leptin and adiponectin fall acutely but may rebound with acclimation; resistin rises in rodents (likely less relevant in humans). Cold-induced browning and mitochondrial adaptations are reversible upon rewarming, highlighting the seasonal plasticity of WAT.

### Liver

**Figure 3.**
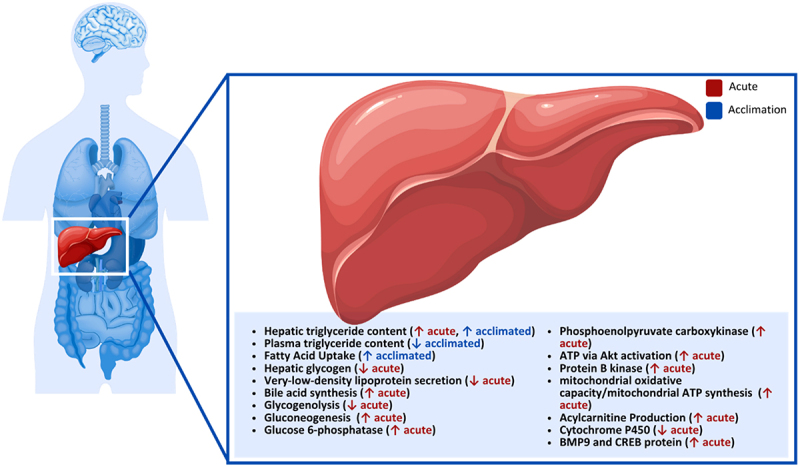
Effect of cold exposure on the human liver.

The liver is a major metabolic organ in the body [46,176], oxidizing triglycerides, synthesizing lipoproteins, phospholipids, cholesterol, bile and nonessential amino acids, and regulating glycogenesis and glycogenolysis (Figure 3). The impact of cold exposure on the liver and hepatic function has been studied in various conditions and states, including acclimation, fasting and non-fasting, and acute and chronic exposures. However, most of these studies have relied on animal-based models [127,177–187]; therefore, the findings must be interpreted as such when considering application to humans.

Changes in hepatic lipids in mice have been observed during short- and long-term cold exposure [177,178]. For example, mice were placed in a climate chamber set at 23°C or 4°C for 24 h or 10 days for short- and long-term protocols, respectively [178]. Hepatic triglycerides increased after 24 h cold exposure, with a decrease in hepatic glycogen and no notable change in hepatic cholesterol [178]. It was also reported that very-low-density lipoproteins secretion decreased, and bile acid synthesis increased due to cold exposure. Following 10 days in the cold, plasma triglycerides progressively decreased as cold exposure continued, and an increase in hepatic triglyceride content was observed [178]. The authors suggested that increased hepatic triglyceride content was likely caused by increased fatty acid uptake from enhanced WAT lipolysis during cold exposure [178]. Further, the effects of cold acclimation and fasting on hepatic lipid and carbohydrate metabolism were examined in rats in control (25°C) or cold (0–2°C) conditions [177]. Half of the rats in each group had access to food, while the other half fasted for 24 h on the final day. Findings demonstrated that liver glycogen content was lower, and lipid content was higher only in the control-fasted rats. In contrast, the control and cold-fed and cold-fasted rats all displayed similar levels of hepatic glycogen and lipids [177]. These results indicated that long-term cold exposure influences hepatic responses to fasting, as the rats maintained hepatic glycogen and lipid levels better than fasted, non-acclimated rats.

Cold exposure also influences glucose metabolism in the liver of rodents [179]. Changes in gluconeogenesis in cold-exposed and cold-acclimated rats were also examined [179]. Based on previously observed increases in glucose turnover, it was suggested that the rate of glucose synthesis also increased. Findings showed that gluconeogenesis is more active in acute cold-exposed rats (1–7 days) compared to long-term cold-exposed rats (3 weeks), implying that gluconeogenesis is not directly related to non-shivering thermogenesis and is essential during shivering to supply the skeletal muscle with glucose to support shivering during initial cold exposure. Later work examined the effects of acute extreme cold exposure (4 h at −15°C) on the liver cells of rats and found lower hepatic glycogen content compared to a control group (~30% from control) [180]. They also observed increased expression of glucose 6-phosphatase (~5 fold) and phosphoenolpyruvate carboxykinase (~2 fold) in the cold-exposed rats, indicating that gluconeogenesis and glucose outputs increased to meet energy demands during cold exposure [180], with a transient increase of cellular ATP produced in the liver. ATP concentration also increased over 1 h (~0.41 *µ*mol·mg^−1^ protein), returning to baseline at 2 h (~0.26 µmol/mg protein), and decreased after 4 h (~0.18 *µ*mol·mg^−1^ protein) in cold-exposed rats. The authors suggested that the increase in ATP was caused by the activation of protein B kinase, a kinase involved in many regulatory signals of cell growth and metabolism [180]. To determine the influence of protein B kinase on ATP concentration, an inhibitor (phosphoinositide 3-kinases, Wortmannin, [15 *μ*g·kg^−1^ body weight]) was injected into rats 1 h before cold exposure. Rats with the inhibitor had a more minor increase of ATP after 30 min and a further decrease after 4 h of cold exposure. Therefore, the activation of protein B kinase by acute cold exposure is essential in maintaining energy balance in liver cells.

Cold exposure and high-fat feeding interactions have been found to influence liver oxidative activity and hepatic metabolism in rats. Cold exposure and high-fat feeding induce similar changes in energy intake associated with alterations in energy expenditure and fuel partitioning; however, only cold exposure increased the mitochondrial oxidative capacity of the liver [181]. Further, cold-exposed rats fed a high-fat diet exhibited the highest T3 levels, displaying additive effects between conditions. Based on these findings, the authors concluded that cold exposure activates mitochondrial ATP synthesis in the liver. In contrast, high-fat feeding induces decreased hepatic efficiency to waste excess ingested energy. This was attributed to the differences in the liver responses to differing hormonal pathways involved in thermogenic stimuli [181].

Moreover, transcriptomic analysis in the liver of mice in addition to BAT and WAT in response to 24 h cold exposure at 8°C was conducted [127]. Of the 1895 genes expressed, only five of these genes (UCP1, PGC-1α, CCAAT/enhancer binding protein, peroxisome proliferator-activated receptor alpha, hepatocyte nuclear factor 4 alpha) were shared by all three tissues. In the liver, the most common direction of change in gene expression was suppression, identifying 590 suppressed genes. In response to the cold, the analysis revealed significant down-regulation of genes involved in oxidoreductase activity, lipid metabolic processes and protease inhibitor activity in the liver of mice [127], and the importance of down-regulation of cytochrome P450 gene expression and apolipoprotein in response to cold exposure. It was proposed that the response to cold stress involves decreased gene expression in various cellular processes to maximize pathways involved in heat production [127].

More recently, strong evidence for the critical role of the liver in providing acylcarnitines as a fuel for non-shivering thermogenesis by BAT in cold-exposed mice has been provided, identifying it as a crucial site for cold adaptation [182]. Cold exposure in rats has been shown to increase hepatic gluconeogenesis, total liver and mitochondrial mass, respiration capacity of hepatocytes, and liver temperature [183–185], providing BAT with glucose and fatty acids from very-low-density lipoproteins, and contributing to heat generation [185]. It was further demonstrated that acylcarnitines are generated from enhanced hepatic fatty acid oxidation in response to cold-induced fatty acid release by WAT [182]. Recently, a mouse model study has shown that the liver plays a more central role in thermogenesis than previously thought. This study demonstrated that bone morphogenic protein 9, which can accelerate adipocyte browning, is produced after cold exposure due to activation of cyclic adenosine monophosphate response element binding protein [186,p.9].

Although BAT is more abundant in rodents than humans, the findings suggest potential benefits for humans in manipulating the adaptive capacity of the liver or providing the thermogenic fuel L-carnitine to increase energy expenditure. However, further human studies are needed to assess the potential role played by the liver. In summary, cold exposure affects hepatic function in various capacities, including hepatic glycogen, lipid levels, fuel utilization and gene expression across multiple exposure lengths and acclimation states. There is also evidence that hepatic glycogen levels are maintained when exposed in a fasting state, glucose synthesis rates increase in acute cold exposure, and hepatic glycogen significantly decreases following cold exposure. Further, it was determined that gluconeogenesis increases but is not directly related to non-shivering thermogenesis and is more critical during shivering. Cold exposure has also been shown to increase hepatic triglyceride content, decrease very-low-density lipoprotein secretion and increase bile acid synthesis while allowing the maintenance of lipid levels in a fasting state. Evidence of transient increases of ATP in the liver caused by the activation of protein B kinase during acute cold exposure has been presented, along with its role in maintaining energy balance in liver cells. Lastly, there is evidence that over 500 genes are suppressed in response to cold exposure and are involved in the liver’s oxidoreductase activity, lipid metabolic processes, and protease inhibitor activity.

**Table:** 

**Key Highlights of the Effect of Cold Exposure on the Human Liver** Cold exposure reshapes hepatic fuel metabolism, with increased gluconeogenesis and reduced glycogen stores during acute cold. Hepatic triglycerides accumulate in cold, while VLDL secretion decreases and bile acid synthesis increases. Liver helps sustain whole-body energy needs during fasting under cold conditions by maintaining glycogen and lipid balance. Acylcarnitine production from hepatic fatty acid oxidation supplies BAT as a thermogenic fuel. Transcriptomic studies show suppression of >500 genes in lipid and oxidoreductase pathways, highlighting systemic reprioritization under cold stress. Emerging evidence suggests the liver functions as a central coordinator of thermogenesis, though human data remain limited.

### Kidneys

**Figure 4.**
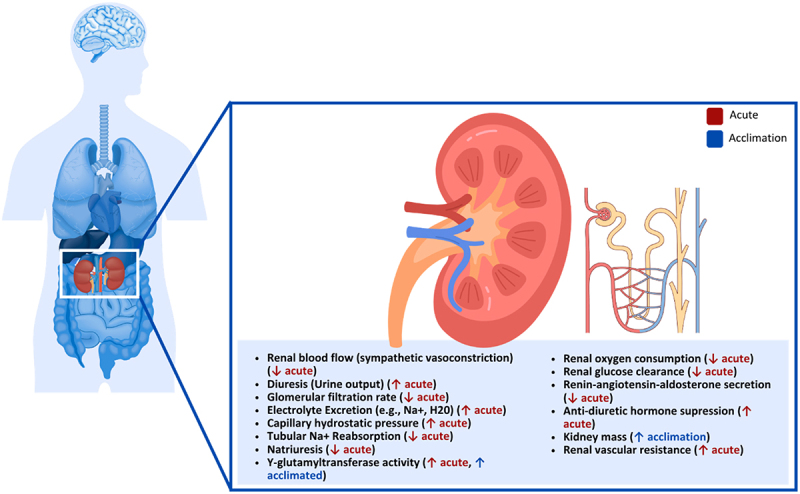
Effect of cold exposure on human kidneys.

The kidneys function as both an excretory and endocrine organ (Figure 4) [187,188], which plays an essential role in systemic metabolism [189], including generating glucose through gluconeogenesis, clearing insulin from circulation, performing critical steps of the urea cycle and regulating the metabolism of fat-soluble vitamins [190]. Early studies suggested that decreases in cutaneous blood flow, induced by external cooling, are accompanied by increased blood flow to visceral regions in humans [191,192]. However, human and animal studies have shown that the increase in visceral blood flow does not occur uniformly among organs [193–195]. In particular, renal blood flow is consistently reduced during cold exposure, despite systemic blood pressure often being elevated due to peripheral vasoconstriction [193,196–198]. This reduction in renal perfusion is likely mediated by sympathetic vasoconstrictive mechanisms and can contribute to impaired metabolic processes within the kidney [199]. Although little human research has been conducted on the kidneys’ impaired metabolic function due to cold exposure, a series of case reports and animal studies have provided an initial basis for understanding the impact.

Cold-induced diuresis is the most established consequence of cold exposure in humans [200,201], which can occur before core cooling. Early observations identified that kidney filtration increases, and urine production happens when a cold stimulus is present. Additionally, when cold exposure occurs after a warm exposure, higher urine output occurs than when cold exposure is first [202]. A similar increase in diuresis (163%) was observed with cold water immersion, where participants submerged below the neck in 14°C water for 1 h compared to warmer water (20°C, 32°C) [203]. Some authors suggest that cold-induced diuresis is an autoregulatory response of the kidney relative to central hypervolemia (fluid overload) induced by peripheral vasoconstriction and elevated blood pressure. In humans, increased central blood volume suppresses the release of antidiuretic hormone, resulting in decreased blood volume and progressive hemoconcentration [200]. Another suggested mechanism is that cold-induced diuresis may be caused by osmotic alteration in the renal tubules, where renal function is depressed due to a fall in systemic blood pressure and the cold’s indirect effect on organ metabolism. Increased renal excretion of sodium and water is another well-known finding in acute cold stress [204], suggesting that cold exposure increases the sympathetic nervous system and raises arterial blood pressure through an increase in cardiac output, thereby increasing capillary hydrostatic pressure in some vascular areas of the human body [205]. It has been postulated that this negatively affects capillary reabsorption processes in the kidney, which causes a reduction in tubular sodium reabsorption and natriuresis. Authors suggest that the decrease in tubular reabsorption of sodium under acute exposure to cold is the balance between hydrostatic and oncotic pressures in the peritubular capillaries, mainly due to increased arterial blood pressure [205].

Numerous authors have also reported that severe cold exposure increases renal blood flow in human participants and experimental animal studies [193–195,205]. For example, a study of tissue blood flow distribution during a cold-induced thermogenesis activation protocol in conscious warm- and cold-acclimated rats was conducted where blood flow to the kidneys was unaffected by cold exposure compared to relative changes between other tissues and organs [206]. In both acclimated groups, the fractional distribution of cardiac output to the kidneys declined with a reduced ambient temperature. In contrast, the fractional distribution of cardiac output to the heart, ribcage and diaphragm increased. However, kidney blood flow was higher in the cold- versus warm-acclimated rats at all temperatures (except 25°C), but was hypothesized to be due to the greater intestinal absorption of nutrients and greater waste disposal (i.e. urea nitrogen), which are required when living at 6°C to consume more food than rats living at 28°C. The effects of acute and chronic stresses, including cold stress, on y-glutamyltransferase activity in rat kidneys has also been investigated and found that y-glutamyltransferase, a transferase enzyme known to regulate reabsorption of amino acids from the urine to the blood, was significantly increased in cold-adapted rats [207].

In hypothermic conditions, renal oxygen consumption is more severely reduced relative to other organs [201] and can result in the clinical presentation of mild/acute and severe renal insufficiency and/or failure in humans [200,208]. During mild hypothermia (32–35°C), cold-induced diuresis occurs before any fall in body temperature, initially due to increased renal blood flow consequent of vasoconstriction. As body and ambient temperatures drop, distal tubular reabsorption of water is reduced, and resistance to the action of antidiuretic hormone (human vasopressin) occurs. The cold-induced diuresis is accompanied by increased urinary electrolyte excretion, likely due to reduced tubular sodium reabsorption [209–211]. In moderate hypothermia (28–32°C), the glomerular filtration rate falls as cardiac output and renal blood flow are reduced [210]. There is also a further reduction in tubular function and renal clearance of glucose, increasing the risk of renal tubular acidosis and acute kidney injury. Lastly, with severe hypothermia ( < 28°C), oliguria (production of abnormally small amounts of urine), and acute renal failure [212] and tubular necrosis [213] can occur.

Cold exposure has also been associated with increases in renin secretion from human kidneys, stemming from increased sympathetic tone and decreased renal blood flow [214]. When the kidneys release renin, it interacts with angiotensin to stimulate the release of aldosterone, resulting in the reabsorption of water and salt to maintain fluid balance [188]. The effects of cold exposure on these hormones in humans were observed and found that during whole-body cooling, plasma renin activity decreased significantly with moderate cold exposure (4°C air for 1 h), and angiotensin II decreased insignificantly [215]. In contrast, plasma renin activity and angiotensin concentrations were not affected when exposed to severe cold with hands immersion in 0°C water for 10 min [215]. It has previously been reported that an increase in plasma renin activity occurs in response to the cold; however, this finding is not consistent with other human studies [214–216]. When participants were submerged below the neck in 14°C water for 1 h, plasma renin activity decreased, and plasma aldosterone concentration increased, indicating that the strength of a cold stimulus may also influence plasma aldosterone [203]. Repeated cold water immersion of participants in 14°C water three times a week for 6 weeks did not affect plasma renin activity, as activity decreased by the same amount before and after acclimation [11]. The authors also reported a progressive increase in plasma aldosterone as acclimation progressed; however, these changes were insignificant.

Although there is a clear relationship between cold exposure, decreases in plasma renin activity and increases in plasma aldosterone, the changes in aldosterone are not likely caused by changes in the renin-angiotensin system. Instead, it was hypothesized that in humans these changes are due to stress-induced adrenocorticotropic hormone secretion [203]. Still, previous research observed no significant increase in adrenocorticotropic hormone concentration in response to cold air [203]. As such, research is needed into the mechanisms of increased plasma aldosterone caused by cold exposure. More recently, the effect of prolonged water temperature during whole-body immersion on plasma volume and hydromineral homeostasis was assessed. The authors found that the participants plasma volume decreased, with cold conditions accentuating the change [217]. Specific to the kidney, the authors found that diuresis during immersion was 2.5–3 times higher, with increased glomerular filtration rate, reduced tubular sodium reabsorption, and lower free water reabsorption in the collecting duct [217]. It was concluded that the adaptive changes in renal function during whole-body immersion (adjusting blood volume and circulatory hemodynamics) are modulated according to increases in cold-induced vasoconstriction or decreases by sweating volume loss [217].

In summary, cold exposure affects renal function in various capacities, with the most known effects being a reduction in renal blood flow and cold-induced diuresis. Although well-established, the mechanisms of cold-induced diuresis are debated. Some authors suggest that the response is relative to central hypervolemia and induced by peripheral vasoconstriction and the elevation of blood pressure. Others suggest it is caused by osmotic alterations in the renal tubules or changes in antidiuretic hormone (human vasopressin) secretion. Additional impacts on the kidney include increases in renal vascular resistance, decreases in glomerular filtration, increases in the excretion of sodium, a change in the balance between hydrostatic and oncotic pressures in the peritubular capillaries, and adverse effects on capillary reabsorption processes in the kidney. Further, acute cold exposure has been found to decrease plasma renin activity and angiotensin II; however, the strength of the cold stimulus was found to influence concentrations and plasma aldosterone as acclimation progresses.

**Table:** 

**Key Highlights of the Effect of Cold Exposure on Human Kidneys** Cold-induced diuresis is the most established renal response, occurring even before core cooling. Cold reduces renal blood flow via sympathetic vasoconstriction, despite elevated systemic blood pressure. Natriuresis and electrolyte loss result from impaired tubular sodium reabsorption and altered capillary pressures. With progressive hypothermia, renal function declines (i.e. increased diuresis [mild] to reduced GFR and clearance [moderate] to risk of acute kidney injury and tubular necrosis [severe]). Cold stress alters the renin-angiotensin-aldosterone system, though findings are inconsistent and stimulus-dependent. Kidneys contribute to systemic cold adaptation by regulating fluid balance, blood pressure, and metabolic waste clearance, but may become vulnerable in prolonged or severe exposures.

### Heart

**Figure 5.**
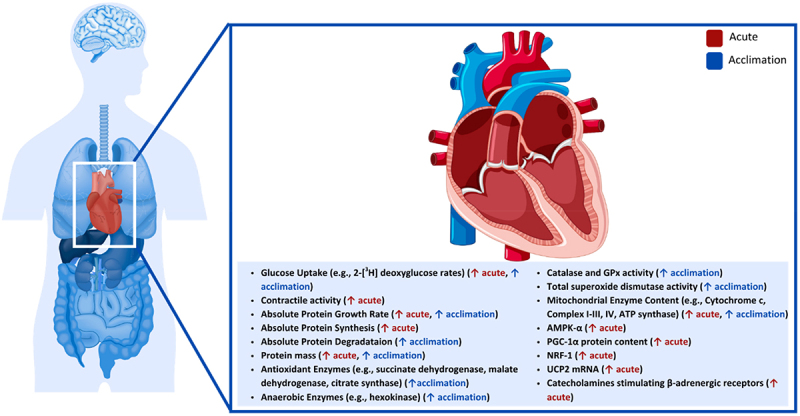
Effect of cold exposure on the human heart.

In cold environments, glucose uptake is increased in the cardiac muscles of rodents (Figure 5) [73,218]. During acute exposures (48 h at 4°C), uptake of 2-[^3^H] deoxyglucose rates are increased to a similar magnitude to skeletal muscles and BAT in rats, with the heart having the second highest uptake rate after BAT [73]. Similar results were observed in another rat study following a 48 h cold exposure and a 3 week acclimation period at 5°C, where glucose uptake was increased 8 times and 15 times, respectively, compared to a control group [218]. Notably, glucose uptake was similar to the control group when rats were reintroduced to a thermoneutral environment (25°C) for 18 h following the cold acclimation [218]. Compared to other tissues, the heart was the only tissue that increased glucose uptake during acclimation. This could be related to increases in heart rate during cold exposure, indicating the importance of glucose in contractile activity over thermogenesis [218].

Protein turnover in the heart also increases during short-term cold exposure and acclimation of rodents [219,220]. The effects of short-term cold exposure were examined by housing rats for 5 days at 5°C and a 5-day rewarming period at 25°C [220]. During the cold exposure, the absolute protein growth rate increased by 82% and 75% after day 2 and day 5, respectively [220]. The absolute protein synthesis was increased by 53% after day 2 and 58% after day 5, while absolute protein degradation was 50% greater compared to control conditions throughout cooling [220], suggesting a greater turnover, but also a greater protein mass buildup. The authors suggested that the cold exposure and its increases in central and cardiac workload would be responsible for this cardiac protein accretion. During the rewarming period, protein growth decreased by 70% after 2 days and 36% after 5 days compared to control rats, while protein synthesis and protein degradation were not different compared to control conditions [220]. Similar changes in cardiac protein turnover occurred during a 20-day cold acclimation period (5°C) of rats [219]. Following the acclimation period, absolute cardiac protein synthesis rates increased by 44%, while protein degradation increased by 54% compared to control conditions [219]. Protein mass increased throughout acclimation, resulting in 28% greater mass and an absolute growth rate 22% higher than control conditions [219]. Interestingly, cardiac muscle protein mass was still increasing at the end of the acclimation period, suggesting that acclimation, and potentially cardiac peak mass had not been achieved during the 20 days. This could explain why protein synthesis and degradation remained elevated in the cold-exposed rats [219]. However, continuously elevating heart protein mass growth is not sustainable as it mimics the pathology of hypertrophic hearts [219]. However, the increase in protein growth and turnover during cold exposure must increase heat production as elevated oxygen and substrate delivery, as well as increased contractile activity requires repair or replacement of damaged proteins [219,220]. Although it is unknown how heart metabolism contributes to thermogenesis, it would seem to be highly regulated by acute and chronic cold exposure.

Various enzymes related to energy production are also altered within the heart tissue of animals during exposure to cold stimuli [221,222]. Enzymes involved in aerobic oxidation have been shown to increase in rat cardiac muscles following a 5-week cold acclimation at 4°C [221], including succinate dehydrogenase (41%), malate dehydrogenase (23%) and citrate synthase (15%), while the anaerobic enzyme hexokinase also increased (15%) [221]. Similar changes were observed during exercise compared to cold exposure. Therefore, it is likely that increased enzyme activity is caused by the release of catecholamines stimulating β-adrenergic receptors and resulting in aerobic tissue metabolism [221]. Enzymes involved in removing reactive oxidative species during aerobic respiration in small mammals were increased in voles that were born and maintained at 8°C for 61 days [222]. In the cold-exposed voles, catalase and selenium-dependent glutathione peroxidase activity increased by 40% and 43%, respectively. In comparison, total superoxide dismutase activity increased 22% but was insignificant from control conditions [222]. Increased activity of these enzymes could indicate increased metabolic rate and reactive oxidative species production.

Furthermore, proteins related to mitochondria energy production are altered depending on the duration of cold exposure. Acute cold exposure and cold acclimation were used to examine the changes in protein content in squirrels undergoing a 21 day cold acclimation at 4°C [223]. Increases in mitochondrial content of the electron transport chain and ATP synthase were observed after various durations of cold exposure, with complex IV, cytochrome c and ATP synthase rising after 7 days and complex I, II and III increasing after 21 days [223]. The expression of energy regulators in the rodents were also altered, with AMP-activated protein kinase catalytic subunit alpha increasing after days 1 and 3 but then returning to control levels the following days, and PGC-1α protein content increased every day up to day 7 [223]. However, citrate synthase activity remained unchanged compared to control conditions, indicating that cold exposure did not affect mitochondrial content [223]. Similar findings were recently published, where after cold exposure, a significant increase in the expression of PGC-1α and NRF-1 was found, which might be due to the rise of silent information regulator sirtuin 1 activity [224].

Moreover, it was also demonstrated that the expression of UCP2 in cardiac muscles could be influenced by cold exposure, as the expression of UCP2 was increased 4.3-fold after mice were exposed to 48 h of cold conditions compared to control conditions [225]. Due to similar expressions of UCP1 and UCP2 in BAT, it has been suggested that similar mechanisms modulate its expression in cardiac muscle [225]. For example, a 2.1-fold increase of UCP2 messenger ribonucleic acid in BAT was reported after a β3-adrenoceptors agonist (Ro-168714) was administered, indicating an increase in SNS modulation of β3-adrenoceptors during preliminary studies [225]. Finally, cold exposure has also been associated with myocardial infarction and arrhythmias in mice, where the cardiac injury is caused by inhibition of the nuclear respiratory factor 2-Kelch-like ECH-associated protein 1 signaling pathway [226]. According to a recent meta-analysis, a decrease of 1°C was associated with a 1.6% increase in cardiovascular disease-related mortality and a 1.2% increase in morbidity [227].

**Table:** 

**Key Highlights of the Effect of Cold Exposure on the Human Heart** Cold exposure increases cardiac glucose uptake, sometimes exceeding most tissues (except BAT). Protein synthesis and degradation rise during cold, leading to higher protein turnover and cardiac mass accretion. Chronic cold exposure induces enzyme and mitochondrial adaptations, enhancing oxidative capacity and antioxidant defences. UCP2 expression is upregulated, suggesting parallels to BAT thermogenic pathways. While adaptive in the short term, prolonged cold may impose cardiac hypertrophy-like changes and elevate risk of arrhythmias and cardiovascular disease in vulnerable populations.

### Pancreas

**Figure 6.**
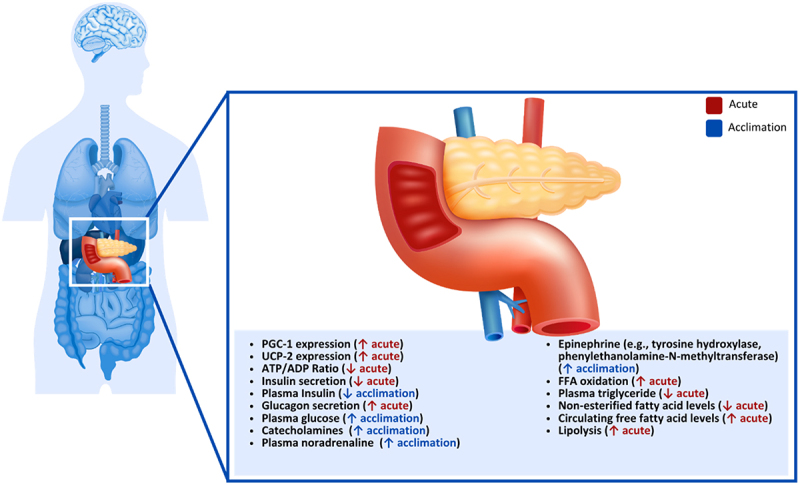
Effect of cold exposure on the human pancreas.

During cold exposure, PGC-1 expression is elevated in humans, which is involved in controlling increases in UCP-2 expression in pancreatic islets, causing reductions in the ATP/adenosine diphosphate ratio in beta cells, subsequently decreasing the secretion of insulin [228] (Figure 6). This effect was observed in rats during acute cold exposure (48 h at 5°C), where plasma insulin concentrations decreased in both fed and starved rats while improving glucose tolerance [71]. These results are contrary to others who observed no changes in plasma insulin in mice following 150 min at −5°C [229]. However, the variation in plasma insulin responses may be associated with the duration of cold exposure since changes in insulin secretion were also observed following cold acclimation. In this study, the acclimatized rats were exposed to 5°C for 4–5 weeks, after which plasma insulin was decreased, but insulin concentrations within the pancreas remained unchanged [229]. Similar observations were made in rats separated into 2-, 4- and 6-week acclimation groups (4°C), which showed continued decreases in plasma insulin concentrations as acclimation progressed, with the 4- and 6-week acclimation demonstrating significant reductions in plasma insulin [230].

In contrast to insulin, glucagon secretion from the pancreas is increased during cooling. In mice following a 150 min cold exposure at −5°C, plasma glucagon concentrations were significantly increased, while glucagon levels within the pancreas did not change [229]. Changes in glucagon secretion have also been observed in humans during acute cold exposure, where glucagon was increased after 90 min of cold exposure (10°C) compared to thermoneutral (23°C) [231]. These changes are also observed in mice following cold acclimation, where plasma glucagon was increased after a 5-week acclimation period; however, there was no change in the glucagon content of the pancreas [229]. Slightly different changes in glucagon secretion were observed following cold acclimation, where rats in 2-, 4- and 6-week acclimation groups (4°C) increased glucagon secretion following the 2- and 4-week cold exposure but then returned to baseline levels during the 6-week cold exposure [230].

These changes in insulin and glucagon have been linked to the sympathetic nervous system increasing catecholamine secretion in humans [29], as increased plasma levels of noradrenaline have been consistently found after cold exposure in rats, rabbits, sheep and humans [232,233,234,235,236,237]. It has also been claimed that increased epinephrine levels in rats can occur seven days after cold exposure by increasing the expression of tyrosine hydroxylase and phenylethanolamine-N-methyltransferase expression, showing non-sympathetic catecholamine production [238]. Norepinephrine seems to have a dose-response effect on both glucagon and insulin secretion in rats, as well as somatostatin, where the more norepinephrine is present, the more glucagon production is stimulated, and the more insulin and somatostatin production is inhibited [239].

Glucagon, which is secreted from pancreatic α-cells, seems to have a stimulatory effect on glycogenolysis and gluconeogenesis, β -β-oxidation of lipids, amino acid transport and urea genesis, while having an inhibitory effect on glycolysis, glycogenesis and lipogenesis in the liver [240]. It also has a stimulatory effect on lipolysis [241]. On the other hand, insulin inhibits lipolysis and glucagon secretion [240], fatty acid oxidation, gluconeogenesis, synthesis but increases de novo lipogenesis, triglyceride esterification, triglyceride secretion [242], glycolysis and glycogen synthesis [243]. The opposing effects of insulin and glycogen support the hypothesis that glucagon determines the actual effect on hepatic metabolism, the insulin ratio and not the absolute concentrations of the hormones [244]. While glucagon levels often increase during acute cold exposure in both animals and humans, changes during cold acclimation are not consistent across studies. This indicates that glucagon responses may depend on exposure duration, acclimation status, and experimental conditions.

Due to the increased glucagon-insulin ratio during cold exposure, stimulation of fatty oxidation and hepatic glucose production can be expected, as suggested by the Randle cycle [245]. This has been observed in goats, where 24 h of cold exposure (6°C) increased plasma glucose levels and decreased plasma triglyceride and non-esterified fatty acid levels [246]. It has also been shown that circulating free fatty acid levels increase during cold exposure in humans [247], which could be explained by glucagon enhancing lipolysis [241].

**Table:** 

**Key Highlights on the Effect of Cold Exposure on the Human Pancreas** Cold exposure alters insulin and glucagon secretion, with insulin often suppressed and glucagon frequently increased acutely. Glucagon changes during long-term acclimation are inconsistent and responses vary by species, exposure duration, and acclimation status. The SNS and catecholamines play a major role, stimulating glucagon and inhibiting insulin release. The glucagon-insulin ratio drives downstream metabolic effects, favouring lipid oxidation and hepatic glucose output. Most detailed findings come from animal models and human data remain limited.

### Bones

**Figure 7.**
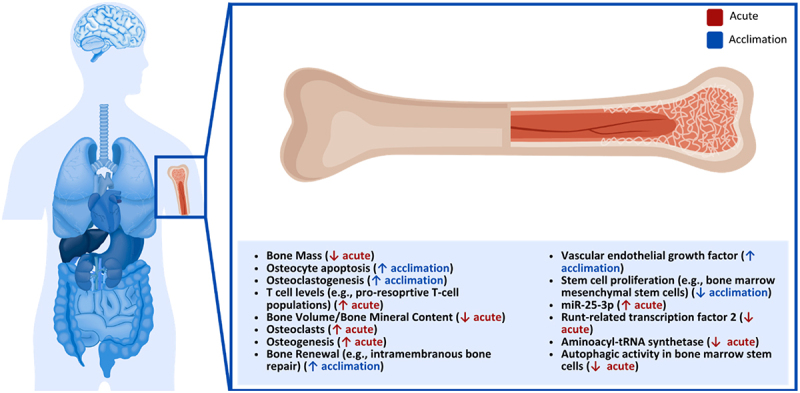
Effect of cold exposure on human bones.

The effects of cold exposure on bone health have recently gained attention in the thermoregulation literature (Figure 7). In an early paper studying rabbits, the effects of cold exposure were only characterized as unfavorable if below freezing [248]. However, with a brief exposure of their feet to either a 0°C or −20°C bath, the rabbits prematurely arrested the piphyseal plate, destroyed the epiphysis and the formation of reactive endosteal and periosteal bone formation, which was thought to be caused by the death of chondrocytes [248]. Since then, it has been shown that cold also has a negative effect on bone loss at above-freezing temperatures in various animals [249–251]. For example, it has been shown that in male mice, exposure to cold temperatures (4°C) compared to normal (23°C) can decrease bone mass after 2 weeks; however, the bone mass recovers after 4 weeks, likely due to osteocyte apoptosis [249]. In another mouse model study, it was shown that even mild cold exposure of 7°C difference (22°C vs 29°C) caused a significant reduction in bone volume and femoral bone mineral content in wild-type mice, while mice lacking neuropeptide Y showed no changes in bone mineral content or volume reduction while increasing energy expenditure [251].

A recent animal model study also found that chronic cold exposure reduces bone mass while enhancing osteoclastogenesis and changing T-cell levels in the bone marrow, like increasing pro-resorptive T-cell populations, which implicates the immune system in the remodeling of the bone [252]. On the other hand, they also found that BAT plays a vital role in protecting against bone loss induced by cold exposure via BAT mitochondrial oxidative phosphorylation [252] and by increasing osteoclast numbers, facilitating osteogenesis *in vitro* and suppressing osteoclastogenesis *in vitro*, which may accelerate bone renewal [253]. This might also be the reason that localized cold therapy has started to be suggested as a bone healing treatment, where daily ice baths were administered to mice for 4-weeks and increased intramembranous bone repair, which has been proposed to be due to upregulation of the vascular endothelial growth factor pathway which led to an increase in osteogenesis [254]. A similar treatment was proposed previously for space travel, where cold exposure treatment (4°C) in a microgravity environment mice maintained muscle strength and bone density [255].

The potential mechanisms of cold on bone, specifically osteoporosis, were recently reviewed [256]. In general, the review found that there is evidence that cold-induced bone loss is caused by the sympathetic nervous system and attenuated by UCP1 in BAT via the hypothalamic neuropeptide Y pathway, but this alone cannot prevent bone loss [256]. A similar finding was reported where UCP-1 upregulation in BAT mitigated but did not completely prevent bone loss in mice [250]. One additional factor is sclerostin, which is significantly changed after cold exposure [98,256]. Various other markers are also crucial in osteoporosis [256]. This includes irisin, which is involved in increasing energy expenditure through UCP-1-mediated thermogenesis and is released after cold exposure, Angiopoitein-like protein, which is increased in BAT and subcutaneous adipose tissue after cold exposure and different versions being involved in various processes promoting osteoporosis, and transient receptor potential channel melastatin 8, which is directly activated by low temperature and upregulates UCP1 expression in BAT [256]. Further, rapamycin, which regulates the serine/threonine kinase machinery and where mechanistic target of rapamycin complex 1 is activated by acute and prolonged cold exposure and mechanistic target of rapamycin complex 2 after mild and severe cold stimulation, and the nuclear respiratory factor 2-kelch-like ECH-associated protein 1 signaling pathway, which is inhibited by cold exposure and regulates bone homeostasis and promotes osteoblast gene expression and osteoblast differentiation [256]. Prolonged cold stimulation (48 h) also lowered the proliferation capacity of bone marrow mesenchymal stem cells [256].

Studies have found a different mechanism with an animal model and studied the effect of prolonged cold exposure on exosomes [257]. They found that miR-25-3p was significantly higher in cold-exposed exposomes and that it reduced osteogenic differentiation by decreasing the expression of runt-related transcription factor 2 and aminoacyl-tRNA synthetase and autophagic activity in bone marrow stem cells [257]. This effect was eliminated by knockout of special AT-rich sequence-binding protein 2, which seems to be a direct target of miR-25-3p and seems to regulate the expression of runt-related transcription factor 2 and aminoacyl-tRNA synthetase, and also by reducing the release of exosomes using the drug N-SMase Inhibitor [257]. Additionally, the effect of miR-25-3p on bone loss was reported to be reduced by treating the cells with antagomiR-25-3p, which seems to reduce the expression of miR-25-3p in bone marrow stem cells [257].

Additionally, a study looked at fourteenth-century inhabitants of Tierra del Fuego, the archipelago in the south of Argentina, due to their exposure to the cold climate without needing clothing [258]. It was found that they had similar bone mineral density as modern humans living in temperate zones, which might be due to Fuegians having a higher frequency of a gene variant that upregulates homeobox protein Hox-C4 expression, which is involved in BAT differentiation and PR domain containing 16, which can increase the activity of BAT by upregulating UCP-1 expression and which has been associated with browning in mouse models when overexpressed [258].

**Table:** 

**Key Highlights on the Effect of Cold Exposure on Human Bone** Cold triggers SNS activity and BAT thermogenesis, affecting bone through hormones, signaling proteins, and exosomal microRNAs. Cold can reduce bone cell viability, slow stem cell proliferation, and alter osteoblast/osteoclast activity, potentially causing temporary bone loss. Brown fat helps protect bone by supporting osteogenesis and partially limiting bone breakdown. In mice, cold can reduce bone mass, but some recovery occurs; genetic factors like UCP1 and neuropeptide Y influence the extent of bone loss. Some human populations exposed to chronic cold maintain bone density, likely due to genetic adaptations that enhance BAT activity, showing systemic protection against cold-induced bone loss.

### Gastrointestinal tract

**Figure 8.**
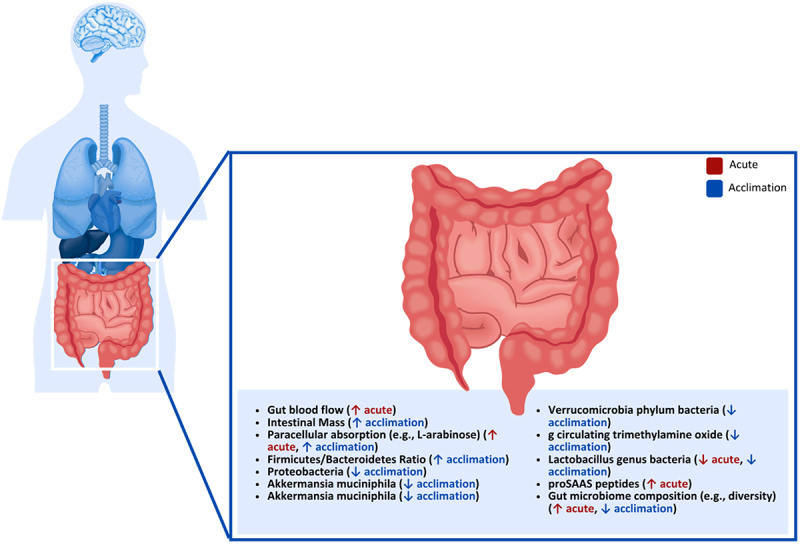
Effect of cold exposure on the human gastrointestinal tract.

The effects of cold exposure on the gastrointestinal tract in humans have received limited attention; however, some research has been performed in animal models (Figure 8). The impact of prolonged cold exposure on nutrient absorption in the small intestine of mice was previously examined using a protocol which exposed them to a 22°C environment for 7 days to acclimate, with a select diet [259]. Half the mice were then moved into a room set at 6°C for 28 days, while the other half remained in the 22°C room for the same time [259]. Food intake increased with the cold-exposed mice with a slight increase in body mass of ~3.6% [259]. It was also observed that while intestinal length remain similar between both groups, the mass of the small intestine was 18%, and large intestine was 20% larger in the cold compared to the room-temperature mice. Interestingly, masses of other organs, such as the kidney and spleen, increased in the cold by 16–20%, whereas the room-temperature demonstrated no noticeable change [259].

It has also been shown that cold exposure influences the paracellular absorption of some nutrients in mice [260]. The immediate and long-term changes caused by cold exposure to the intestinal properties of mice were also examined by observing differences in the fractional absorption of L-arabinose. This carbohydrate is not absorbed by glucose transporter but via the paracellular pathway [260]. Mice were injected with a sodium chloride solution containing L-arabinose and then moved from 20°C to a 4°C room, where they remained for two weeks [260]. The mice were then placed in metabolic chambers to collect their urine after differing intervals from 1.5 to 20 h [260]. After a single day of cold exposure, L-arabinose absorption increased by 20% and remained elevated following two weeks of cold exposure, with a peak absorption difference of 33%, compared to the pre-exposure [260].

Recently, there has been a focus on studying the effect of cold exposure on the gut microbiome in animal models. It has been suggested that short-term cold exposure might increase α-diversity, intermittent cold exposure has no effect, and long-term exposure decreases α-diversity [261]. A similar trend was seen with the composition of the gut microbiota, where short-term and intermittent cold exposure slightly changed the composition of the gut microbiome. In contrast, extreme cold has increased the Firmicutes/Bacteriodetes and decreased Proteobacteria in rats [262]. The change in the F/B ratio could be interpreted as a change intended to maintain metabolic homeostasis by increasing the energy metabolism, in which Firmicutes phylum bacteria are involved. In contrast, the Bacteriodetes phylum bacteria are mainly engaged in degrading carbohydrates and proteins [261]. Verrucomicrobia phylum bacteria, which have been linked with insulin sensitivity, are also nearly wholly removed after cold exposure [261]. A specific *Verrucomicrobia* phylum species, namely *Akkermansia muciniphila*, and g circulating trimethylamine oxide levels, show lower abundance during cold, potentially affecting atrial fibrillation inducibility [263]. The abundance of Firmicutes phylum bacteria of genus Ruminococcaceae and Actinobacteria phylum bacteria of genus Adlercreutzia are upregulated after cold exposure, positively impacting bile acid and energy metabolism and influencing diet-associated obesity [263]. Fluctuating ambient temperature levels between 10°C and 25°C in mouse models have been shown to aggravate muscle atrophy, and this effect could be seen in mice that received a fecal microbiota transplant from the mice exposed to the ambient temperatures [264].

A link between the gut microbiota and the brain during cold exposure has been proposed recently after mice exposed to cold exhibited an altered brain peptidome [265]. The abundance of the *Lactobacillus* genus bacteria was significantly decreased compared to room temperature in acute and chronic cold conditions [265]. Lactobacillus was positively correlated with peptides originating from the proprotein convertase subtilisin/kexin type 1 inhibitor protein precursor following cold exposure, which is associated with energy metabolism [265]. These peptides are thought to play a part in regulating food intake and body weight [266]. After oral administration of *L*. *fermentum* spp. in mice, the abundance of these proprotein convertase subtilisin/kexin type 1 inhibitor family peptides was increased in the hypothalamus, seemingly confirming that Lactobacillus influence neural peptides [265]. Additionally, treatment with cold-adapted microbiota in mice seems to decrease hypothalamic neurokinin B, contributing to a change from lipids as an energy source to glucose [265]. Excessive monosaccharides might activate immune cells, leading to insulin resistance and inflammation. Alistipes indistinct provides an interesting potential treatment for insulin resistance, showing that gut microbial carbohydrate metabolism might play an essential role in insulin resistance [267]. However, as stated earlier, the gastrointestinal tract is still actively researched, and human studies are limited. Therefore, whether or to what extent these findings are relevant to human physiology is unclear.

**Table:** 

**Key Highlights on the Effect of Cold Exposure on the Human Gastrointestinal Tract** Most findings come from animal models, as evidence in humans is limited. Cold exposure increases food intake and intestinal mass in mice, supporting greater nutrient processing. Paracellular absorption of carbohydrates rises acutely and remains elevated with chronic cold. Cold alters gut microbiome composition (i.e. short-term exposure may increase diversity, but prolonged cold reduces it). Increased Firmicutes/Bacteroidetes ratio and depletion of Akkermansia muciniphila suggest shifts in energy metabolism and insulin sensitivity. Cold-adapted microbiota influence hypothalamic peptides and may alter energy substrate use. The link between the gut and brain is implicated in cold adaptation, but human evidence remains limited.

### Brain

**Figure 9.**
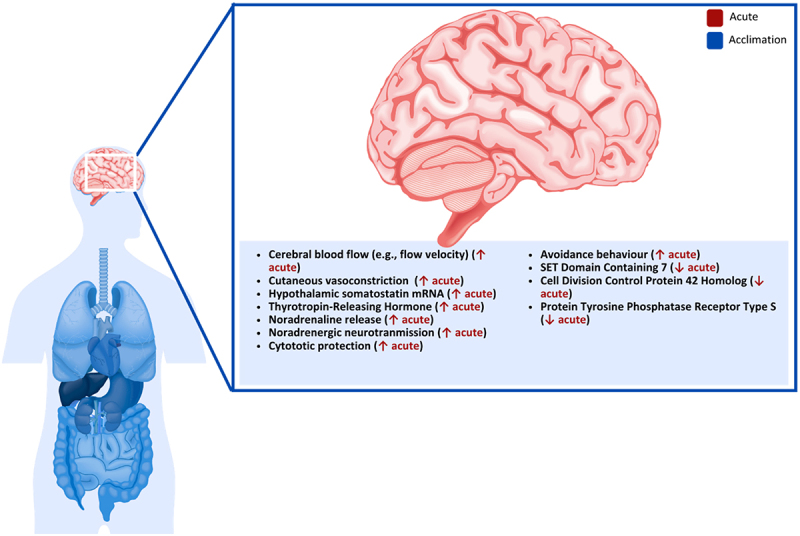
Effect of cold exposure on the human brain.

While whole-body core temperature is usually maintained within a narrow range despite changes in ambient temperature, brain temperature can fluctuate by up to ~3°C under normal physiological conditions [268,269] (Figure 9). In healthy individuals, brain temperature rarely falls below 34–35°C or rises above ~40°C, due to tight regulation of cerebral blood flow and heat exchange mechanisms. This range helps protect neural tissue from the potentially harmful effects of more extreme temperature shifts that can occur in peripheral tissues. However, temperatures exceeding ~40°C can rapidly cause damage to the brain cells [268].

The brain’s temperature depends mainly on brain metabolism and cerebral blood flow [270,271]. The arterial blood arriving from the core body is approximately 0.2°C lower in temperature than that of the brain, and increases in cerebral blood flow remove excessive heat from the tissue [272]. Facial and head cooling studies have been employed to examined brain blood flow regulation under thermal stress. A 60-second cold stimulus to the forehead increased mean medial artery flow velocity by 5.4 cm s-1 in 17 healthy male and female subjects [273]. The observed increase in flow velocity was speculated to arise due to the diving reflex. Similarly, low-intensity exercise, combined to face cooling for 3 min with cold (4°C) mist also induced cerebral blood flow in healthy men [274]. Ice cream headache (or brain freeze) also increases the mean flow velocity in the middle cerebral artery in healthy volunteers [275]. The regulation of brain temperature and its maintenance has also been suggested to be influenced by upper airways heat loss during ventilation [276], but other key mechanisms such as alteration of the blood-brain barrier have also been suggested in rodents [277]. Within a physiological range of ~30–36°C, studies have shown a protective effect of cold on the blood-brain barrier in rodents, suggesting a cytotoxic protection against edema or ischemic injuries [201,278]. Although cerebral blood flow may increase under cold stress to maintain brain temperature, increasing the tightness of gap junctions of the blood-brain barrier limits the sudden increase in fluid and solutes to protect brain tissues.

Ambient cold exposure (8°C) in rats was reported to increase hypothalamic somatostatin levels, metabolism and cutaneous vasoconstriction in rats after 90 min [279]. Hypothalamic somatostatin, along with thyrotropin-releasing hormone, is involved in regulating the release of pituitary thyrotropin. The messenger ribonucleic acid levels of both somatostatin and thyrotropin-releasing hormone have been shown to acutely increase under cold stress (4°C, 15 min), but seem to return to control values at 30 min in rats [280]. It has been proposed that the increase in plasma thyrotropin from cold exposure is more pronounced with a greater temperature changes, where male rats acclimated to 30°C exhibited higher levels of plasma thyrotropin than rats acclimated to room temperature [281].

Intermittent cold exposure has also been found to induce changes in the protein expression profile in the hypothalamus and pituitary using proteomics validated with enzyme-linked immunosorbent assay, where Cell Division Control Protein 42 Homolog and Protein Tyrosine Phosphatase Receptor Type S (PTPRS) levels, as well as the activity of SET Domain Containing (Lysine Methyltransferase) 7, were reported to be significantly reduced in female rats [282]. Cell Division Control Protein 42 Homolog belongs to the Rho family of small guanosine triphosphatases and was reported to have a part in regulating the actin cytoskeleton via Cell Division Control Protein 42 Homolog interacting protein 4 [283], hindering the integrin-dependent T cell-dependent antibody response, germinal center formation and integrin-mediated recruitment of T cells to cutaneous sites via interacting protein 4 if missing [284], responding to pathologic stress in cardiac myocytes via interacting protein 4 [285] and being involved in various tissues for other functions [286]. Protein tyrosine phosphatase receptor S has been reported to have a protective effect against tubulin-associated unit pathology and synaptic destruction in human males [287], protecting against cancer [288], restricts the activation of human and murine plasmacytoid dendritic cells [289], SET Domain Containing (Lysine Methyltransferase) 7, a protein methyltransferase, is involved in promoting tumor promotion, promoting cell cycle arrest and apoptosis [290].

Additionally, it has been suggested that exposure to cold environments may induce negative emotions due to the significantly increased release of noradrenaline in the ventral bed nucleus of the stria terminalis in rats, increasing noradrenergic neurotransmission [235]. This has been theorized to be a driver of avoidance behavior. In another paper on mice, it was also proposed that the pathway from the paraventricular thalamic nucleus to the ventral bed nucleus of the stria terminalis, nucleus accumbens and central amygdala, which is thought to mediate the approach-avoidance response, was activated after cold exposure [291].

**Table:** 

**Key Highlights on the Effect of Cold Exposure on the Human Brain** Brain temperature can fluctuate by ~3°C despite stable core temperature, but rarely falls below 34–35°C or rises above 40°C. Cerebral blood flow plays a central role in protecting brain temperature; facial/forehead cooling and even “brain freeze” increase middle cerebral artery flow. Based on animal studies, cold may tighten the blood-brain barrier and offer cytoprotective effects against edema or ischemia. Hypothalamic neuropeptides and pituitary outputs are dynamically regulated by cold, especially in rodents. Proteomic changes in hypothalamus/pituitary suggest broader neuroendocrine and stress-adaptive responses. Cold exposure may influence emotional/motivational circuits, with increased noradrenergic neurotransmission linked to avoidance behaviours.

### Metabolically relevant endocrine response

**Figure 10.**
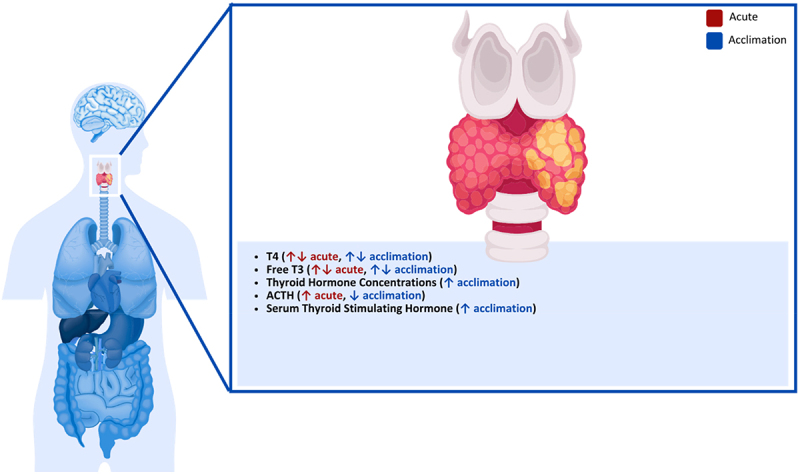
Effect of cold exposure on the human thyroid.

The effects of cold exposure on hormonal secretion are well characterized in humans [16]. Human thyroid hormones regulate energy homeostasis [292], but their role in regulating whole-body temperature remains unclear (Figure 10). Acute exposure to cold does not induce changes in humans’ thyroid-stimulating hormone (TSH) or serum thyroid hormones. However, others demonstrated that serum levels of thyroid-stimulating hormone were higher in humans in December compared with the other months, though the effect was likely attributed to factors other than temperature changes (i.e. photoperiodicity) [58]. A study demonstrated that cold-induced thyroid activation in old male rats was independent of thyroid-stimulating hormone, although free T3 and T4 levels were significantly increased [59]. It was speculated that SNS activation might have had a role in thyroid activation. When considering the effect of chronic cold exposure on thyroid hormones, the results are contradictory. Another study on male infantry soldiers working in interior Alaska found that serum total T4 and T3 concentrations increased in the winter, while free T4 and T3 concentrations were the highest during spring [275].

Thyroid hormone concentrations in male soldiers working outdoors 6–10 h per day in northern Russia (62°N) have also been studied [293]. The total T4 levels were the highest in August and lowest in March [293]. The free T4 concentrations peaked in September and April. Total serum T3 peaked in December, while the lowest values were measured in April [294]. Serum-free T3 levels were the lowest in June and July [295]. On the other hand, thyroid hormone concentrations in humans have also been reported to decrease due to chronic cold exposure [58,295,296]. For example, serum total T3 and T4 levels were reduced in euthyroid cold factory workers [295]. In another study, total and free T3 levels decreased in male subjects living in Antarctica [296]. In another investigation, blood samples were collected every two months and analyzed the circulating thyroid hormones from 20 healthy males living in Northern Finland (69–70°N) [58]. The free T3 concentrations were the lowest in February, and the levels correlated to the cold outdoor temperature [58]. The hormone consumption induces a drop in serum-free T3 levels, which is called a *“Polar T3 Syndrome”* [58,297]. Thyroid hyperactivity during chronic cold adaptation was also observed in males and females living in Greenland [294]. Recently, it was shown that restoring normal thyroid function in hypothyroid patients results in increased cold-induced thermogenesis [292]. Chronic cold exposure leads to low levels of total and free T3. On the other hand, the observed increases in thyroid hormone concentrations in some studies might be caused by shorter exposure times.

**Figure 11.**
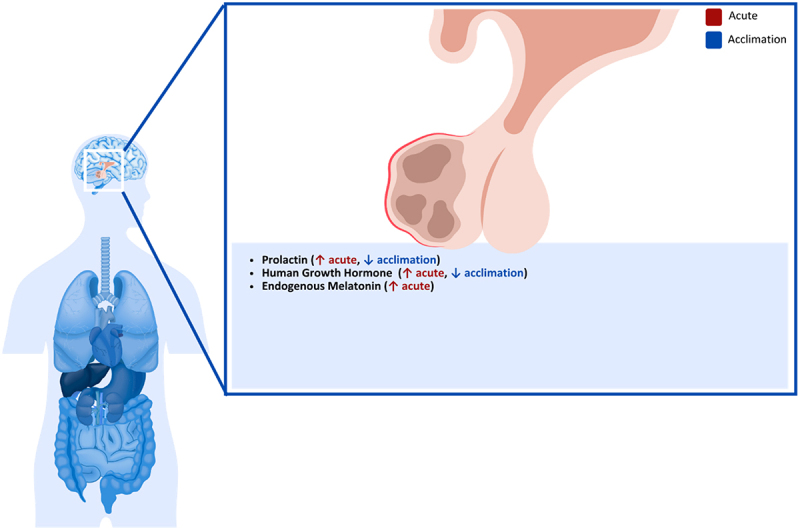
Effect of cold exposure on the human pituitary gland.

It has been demonstrated that 60 min of cold exposure at 10°C increases plasma adrenocorticotropic hormone concentrations in healthy males [298]. In another study, of females, long-term whole-body cold exposure with cryotherapy led to decreased plasma adrenocorticotropic hormone in week four compared with week one [299]. The observed decrease was suggested to be a habituation response to the actual experiment [299]. Cold exposure also affects some other pituitary hormones (Figure 11), such as human growth hormone and prolactin levels decrease under severe cold [16,300]. Although less studied in humans, melatonin levels may also be impacted by cold exposure. An investigation of male subjects exposed them to different illumination intensities and exposure to a cold environment (15°C) 2 h before sleep to suppress melatonin [301]. The cold did not affect salivary melatonin levels. However, it was demonstrated that after cold exposure, high endogenous levels of melatonin correlated with decreased rectal temperature [301]. Thus, melatonin seems to control thermoregulatory processes in the human body. Of note is that melatonin also appears to affect the induction of browning of WAT and the activation of BAT in rats [302,303]. More research is required to study the effects of cold exposure on melatonin levels.

**Table:** 

**Key Highlights on the Effect of Cold Exposure on Metabolically Relevant Endocrine Responses in Humans** Thyroid hormone responses to cold are inconsistent (i.e. acute cold often shows no change, while chronic or seasonal exposures may lead to both increases and decreases). Chronic cold exposure in extreme environments (e.g. Arctic, Antarctica) often reduces circulating free T3, reflecting increased hormone utilization. Pituitary hormones can increase acutely with cold, but may decrease with habituation (i.e. growth hormone and prolactin levels decline under severe cold). Melatonin is generally unaffected by cold exposure in humans, but higher endogenous melatonin is linked to lower body temperature. Endocrine responses to cold are context-dependent, varying by species, exposure duration, season, and population, with important implications for energy balance and thermogenesis.

## Future directions

As new data emerges regarding the limited contribution of BAT to whole-body heat production (estimation of 1% and possibly less), other organs may contribute more significantly than previously thought to thermogenesis. Since skeletal muscles can increase energy expenditure up to five-fold through shivering thermogenesis and may result in greater than 80% of total heat production, some studies should further investigate the thermogenic contribution of the liver and heart and their high glucogenic turnover and metabolic activity. Based on organ mass and energy turnover estimation in rodent studies, both the heart (8 to 15 times glucose uptake) and the liver (increases of 5 times G6P levels and 70% decrease in hepatic glycogen) could account for the remaining thermogenic activity observed under cold stress, each contributing for approximately 5–10% of total heat production. Although challenging to assess in humans and despite a high degree of cold-induced metabolic regulation, from a thermogenic perspective, the other organs presented in this review, including bones, pancreas, kidneys, the GI tract, brain, and even WAT, may have very limited thermogenic influences under cold stress.

**Figure 12.**
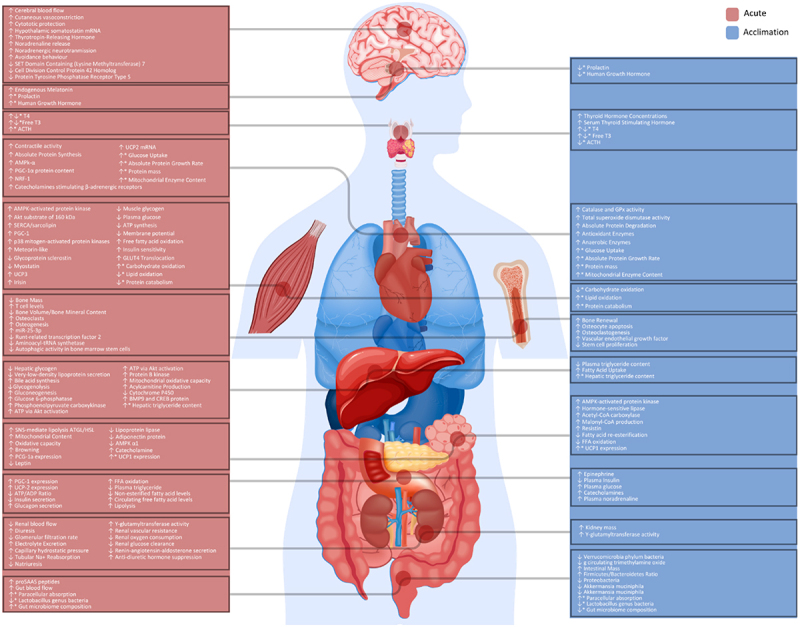
Effect of cold exposure across tissues and organs in the human body.

A major conclusion of this review is that cold exposure affects metabolism far beyond thermogenic fuel partitioning. It influences uncoupling protein activation, gene expression, protein synthesis, hormonal signaling, immune pathways, enzymatic secretion, gut microbiome composition (among others), each of which feeds back into metabolic pathway regulation. However, there was limited literature and a paucity of human studies regarding the metabolism of smaller organs, such as the brain and the gastrointestinal tract, which makes it challenging to determine the extent of their influence on metabolism. Therefore, our presented integrative view underscores the need to move beyond single-tissue studies toward multi-system approaches that can capture the complexity of cold metabolism (Figure 12). Future interdisciplinary research should prioritize a number of novel directions, including clarifying the mechanistic links between cold-induced endocrine and immune modulation and their downstream effects on organ-specific metabolism, determining the long-term systemic consequences of chronic cold exposure in humans (e.g. potential health trade-offs), exploring individualized responses to cold to identify population-specific benefits or risk (e.g. sex, age, genetics, climate adaptation), and evaluating the therapeutic potential of controlled cold exposure for various chronic conditions (e.g. metabolic disorders, obesity, type II diabetes, osteoporosis).

## Conclusion

This review comprehensively presents cold-induced metabolic changes in human organs and tissues. Cold exposure elicits a highly coordinated yet tissue-specific series of metabolic, endocrine, neural, immune and microbiome-mediated responses that together maintain core temperature and energy balance. While cold-induced BAT activation has historically been the focus of thermogenesis research, skeletal muscles remain the most critical organ regulating acute energy expenditure and thermogenesis. This response, however, is short-lived and unsustainable, contrasting with non-shivering adaptations that produce more modest but longer-lasting effects. Therefore, although BAT may still offer interesting approaches to mitigate conditions like obesity and metabolic disorders, a focus on other tissues is warranted. Thus, our synthesis highlights the metabolic regulations of skeletal muscle, WAT, and other metabolically active organs such as the liver, kidneys, heart, pancreas, bones, gastrointestinal tract, brain, thyroid and pituitary gland beyond acute cold exposure and fuel partitioning. The regulation of these responses is not uniform, differing in onset, magnitude and duration depending on the depth, time and mode of cooling. By systematically characterizing how each organ and system contributes to cold stress and adaptation, the field can move toward targeted interventions that harness beneficial metabolic effects while minimizing health risks.

## List of abbreviations

ATP: adenosine triphosphate
BAT: brown adipose tissue
GLUT4: glucose-transporter 4
SNL: sarcolipin
SNS: sympathetic nervous system
SERCA: sarcoplasmic/endoplasmic reticulum Ca^2 +^ -ATPase
T3: triiodothyronine
T4: thyroxine
mRNA: messenger ribonucleic acid
UCP1-3: mitochondrial uncoupling protein 1/2/3
WAT: white adipose tissue
